# Association of structural brain changes with cognitive deficits and fatigue in patients with post-COVID-19 condition

**DOI:** 10.1093/braincomms/fcag099

**Published:** 2026-03-18

**Authors:** Katia Schwichtenberg, Tim Hartung, Josephine Heine, Stephan Krohn, Fabian Boesl, Rebekka Rust, Amy Romanello, Friedemann Paul, Judith Bellmann-Strobl, Christiana Franke, Carsten Finke

**Affiliations:** Department of Neurology, Charité—Universitätsmedizin Berlin, 10117 Berlin, Germany; Department of Neurology, Charité—Universitätsmedizin Berlin, 10117 Berlin, Germany; Department of Neurology, Charité—Universitätsmedizin Berlin, 10117 Berlin, Germany; Experimental and Clinical Research Center, Max Delbrueck Center for Molecular Medicine and Charité—Universitätsmedizin Berlin, 10117 Berlin, Germany; Department of Psychiatry and Neurosciences, Charité—Universitätsmedizin Berlin, Corporate Member of Freie Universität Berlin and Humboldt-Universität zu Berlin, 10117 Berlin, Germany; Department of Neurology, Charité—Universitätsmedizin Berlin, 10117 Berlin, Germany; Humboldt-Universität zu Berlin, Berlin School of Mind and Brain, 10117 Berlin, Germany; Department of Neurology, Charité—Universitätsmedizin Berlin, 10117 Berlin, Germany; Experimental and Clinical Research Center, Max Delbrueck Center for Molecular Medicine and Charité—Universitätsmedizin Berlin, 10117 Berlin, Germany; NeuroCure Clinical Research Center (NCRC), Charité—Universitätsmedizin Berlin, Freie Universität Berlin, Humboldt Universität zu Berlin, Berlin Institute of Health (BIH), 10117 Berlin, Germany; Institute for Immunology, Fatigue Center, Charité—Universitätsmedizin Berlin, 10117 Berlin, Germany; Department of Neurology, Charité—Universitätsmedizin Berlin, 10117 Berlin, Germany; Humboldt-Universität zu Berlin, Berlin School of Mind and Brain, 10117 Berlin, Germany; Experimental and Clinical Research Center, Max Delbrueck Center for Molecular Medicine and Charité—Universitätsmedizin Berlin, 10117 Berlin, Germany; NeuroCure Clinical Research Center (NCRC), Charité—Universitätsmedizin Berlin, Freie Universität Berlin, Humboldt Universität zu Berlin, Berlin Institute of Health (BIH), 10117 Berlin, Germany; Experimental and Clinical Research Center, Max Delbrueck Center for Molecular Medicine and Charité—Universitätsmedizin Berlin, 10117 Berlin, Germany; NeuroCure Clinical Research Center (NCRC), Charité—Universitätsmedizin Berlin, Freie Universität Berlin, Humboldt Universität zu Berlin, Berlin Institute of Health (BIH), 10117 Berlin, Germany; Department of Neurology, Charité—Universitätsmedizin Berlin, 10117 Berlin, Germany; Department of Neurology, Charité—Universitätsmedizin Berlin, 10117 Berlin, Germany; Humboldt-Universität zu Berlin, Berlin School of Mind and Brain, 10117 Berlin, Germany

**Keywords:** post-COVID-19 condition, magnetic resonance imaging, fractals, thalamus, cognitive dysfunction

## Abstract

Cognitive impairment and fatigue are frequent symptoms in patients with post-COVID-19 condition. However, cognitive issues are often only self-reported and not compared to well-matched control groups. Furthermore, structural brain changes, underlying cognitive impairment and fatigue in post-COVID-19 condition are still not fully understood. To assess cognitive deficits, fatigue, neuropsychiatric symptoms and quality of life in patients with post-COVID-19 condition, determine changes in brain volumes, cortical thickness and regional shape complexity and assess correlations of imaging measures with clinical symptoms, 49 patients with post-COVID-19 condition (80% female) with a confirmed SARS-CoV-2 infection at least 3 months prior to testing and new onset of cognitive complaints and 48 healthy controls matched for sex, age and education level underwent comprehensive neuropsychological testing, MRI-based volumetric analyses, and fractal dimensionality analysis to evaluate structural brain complexity. Neuropsychiatric symptoms and quality of life were assessed using questionnaires. Patients with post-COVID-19 condition exhibited significant deficits of attention, executive functions, phonemic and semantic fluency, verbal learning and episodic and visuospatial memory (all *P*(corrected) = 0.002–<0.001). Cognitive impairments were not linked to the severity of the initial COVID-19 infection, but attention performance significantly impacted daily functioning. Patients had a significantly lower quality of life and higher levels of anxiety, depressive symptoms and fatigue compared to controls (all *P*(corrected) < 0.001) and were severely impacted in their ability to work with 45% being unable to work. Thalamic volumes were significantly reduced in patients with post-COVID-19 condition (*P*(corrected) = 0.001–<0.001). Fractal dimensionality analyses showed increased complexity in the occipital lobes and hippocampal fimbriae, and reduced complexity in the thalamus bilaterally in patients. Thalamic complexity reductions correlated with increased fatigue severity in patients and controls. Patients with post-COVID-19 condition display a wide spectrum of cognitive deficits, increased levels of fatigue, anxiety and depressive symptoms and reduced quality of life. MRI analyses revealed reduced thalamic volumes and reduced thalamic complexity that was associated with fatigue severity across the whole sample. These findings identify brain structural correlates of key symptoms in post-COVID-19 condition and highlight the value of fractal dimensionality analysis to detect clinically relevant structural brain alterations that remain undetected in conventional analyses.

## Teaser

Schwichtenberg *et al*. report that patients with post-COVID-19 condition show cognitive deficits across multiple domains, fatigue and reduced quality of life. MRI analyses revealed smaller thalamic volumes and altered brain structural complexity, with thalamic changes linked to fatigue.

## Graphical Abstract

**Graphical Abstract:**
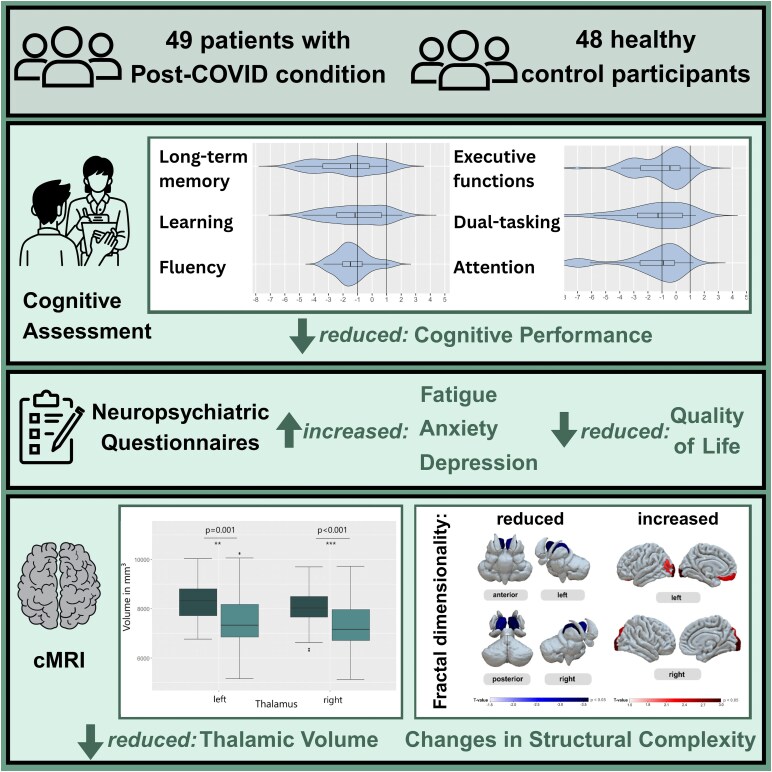

## Introduction

Post-COVID-19 condition (PCC) is defined as persistent or new-onset symptoms occurring at least 3 months after acute COVID-19, lasting for at least 2 months, and not attributable to any other diagnosis.^1^ PCC is estimated to affect up to 10% of patients with COVID-19^2,3^ and can include a wide variety of symptoms including neurological, pneumological, neuromuscular, cardiovascular and dermatological sequelae.^4-6^ These symptoms are associated with reduced quality of life,^7^ inability to return to work^6,8^ and long-term disability. As such, PCC represents a major economic and public health burden.

One of the most debilitating and persistent symptoms is cognitive dysfunction.^5,6^ Early studies found that patients show impairment in memory, attention and executive function.^5,9,10^ However, many of the current studies on cognition in PCC have relevant limitations. First, many studies rely on self-reports of patients, and subjective cognitive assessments do not necessarily reflect cognitive deficits accurately. The full extent of cognitive impairment may not be fully captured and can be influenced by patients’ self-perception, mood and motivation.^11-13^ Therefore, both subjective and objective cognitive assessments are needed to evaluate cognitive deficits. Second, studies with objective cognitive testing in patients with PCC often lack appropriate control groups or they do not exclude patients with pre-existing neurological or psychiatric disorders which can impact their cognitive performance.^14-16^ Considering the psychological impact of the pandemic on society^17^ and the aforementioned impact of psychological conditions on cognitive functioning,^14-16^ it is thus important to compare cognition between patients with and without PCC in a similar pandemic situation. Third, many studies only apply less time-consuming cognitive screening tools instead of detailed neuropsychological testing. While these tools can offer an overview of cognitive deficits, they cannot provide a detailed understanding of cognitive performance. Therefore, studies applying detailed objective cognitive assessments of otherwise healthy patients with appropriate control participants are needed.

The structural brain correlates of neurological PCC symptoms are not yet fully identified. While several studies reported cortical and subcortical grey and white matter changes, results are heterogeneous and often remain inconclusive. One longitudinal study found more severe reduction in grey matter volume in non-hospitalized patients compared to non-COVID-19 control participants or their own pre-COVID-19-MRIs.^18^ These changes in grey matter volume are supported by some studies,^19-21^ while others did not find differences in grey matter volumes between patients with PCC and control participants^22^ or showed increases in some brain regions.^23-25^ Regions repeatedly reported to exhibit changes in grey matter volume include the olfactory cortex, basal ganglia, thalamus and the limbic system.^18-20,24,26^ While most studies observed a volume reduction, volume increases have likewise been reported.^24^ These divergent findings might be driven by differences in patient characteristics and inclusion criteria, as well as different timings of testing in relation to infection. Therefore, studies in well-selected post-COVID-19 patients (i.e. adherence to current diagnostic criteria, exclusion of patients with relevant comorbidities) and healthy controls well-matched for sex, age and education level are needed to clarify the association between neurological PCC symptoms and brain structural changes.

Moreover, sensitive new imaging techniques have emerged to quantify brain morphology from structural MRI beyond standard volumetry. In particular, structural complexity analysis with fractal dimensionality (FD) yields a quantitative account of brain shape, which has proven highly sensitive to both age-related and disease-related changes of brain morphology, including a variety of neuropsychiatric conditions.^27-33^ Briefly, FD stems from a branch of mathematics that quantifies an object’s geometric behaviour across different spatial scales,^34^ which in neuroimaging corresponds to different voxel sizes.^28,35^ Notably, FD can be computed directly from voxel-indexed 3D segmentation masks—the same data used for volumetry—without the need for additional modelling steps or surface reconstructions inherent to many other shape-sensitive methods. Consequently, FD can be flexibly applied to all tissue compartments of the brain using standard T_1_-weighted images. Not least, FD yields improved reliability compared to many classical neuroimaging features^36^ and has been shown to capture morphological information beyond volume and common surface-derived measures including cortical thickness, curvature, sulcation, gyrification and surface area.^27^ FD may therefore offer a valuable addition to neuroimaging analyses in PCC research.

Against this background, the aims of this cross-sectional study were to (i) assess cognitive performance using a comprehensive testing battery; (ii) analyse fatigue, neuropsychiatric symptoms, and quality of life; (iii) evaluate cortical and subcortical volumes, and (iv) apply FD analyses to examine structural brain complexity in patients with PCC and matched non-COVID-19 control participants. With this approach, we aimed to identify cognitive domains with significant impairments in PCC, characterize relevant neuropsychiatric symptom burden, determine changes in subcortical brain volumes, cortical thickness and structural complexity, and investigate whether such brain changes were associated with cognitive impairment in PCC.

## Methods

### Participants and study design

In the prospective observational CAMINO (‘Cognition and MRI in post-COVID’) study, 49 post-COVID-19 patients were recruited between April 2021 and November 2021 from two neurological post-COVID-19 outpatient centres at Charité—Universitätsmedizin Berlin, Germany.^37^ Inclusion criteria for patients with PCC were: (1) positive PCR test for SARS-CoV-2, at least 3 months prior to inclusion, (2) no pre-existing neurological or psychiatric disorders before SARS-CoV-2 infection, and (3) self-reported cognitive impairment with onset during or shortly after COVID-19. The study was exploratory and hypothesis-generating in design and at the time of study planning no reliable data on expected effect sizes were available. We chose the current sample size, since it is sufficient to detect medium-sized effects at a statistical power of 0.8.

Healthy control participants were recruited during the same period via internet advertisements and notes posted at local facilities (*n* = 48). All healthy control participants met the following inclusion criteria: (1) no history of COVID-19 (no positive PCR, no positive rapid antigen test, no reasonable suspicion), (2) no neurological or psychiatric disorders. All participants were 18 years or older, without contraindications for MRI, fluent in German and capable of person-to-person assessments at Charité campus. Patients were matched 1:1 to healthy controls based on age, sex, and years of education using exact matching. The median age was 44.0 years (IQR 17) for patients and 42.5 years (IQR 21) for controls; 80% of patients (39/49) and 81% of controls (39/48) were female; and the median years of education were 16.0 (IQR 5) and 17.0 (IQR 3), respectively. No exclusions were necessary, since there were sufficiently many suitable controls.

Originally, 50 patients and 50 healthy controls were tested for the CAMINO study. However, one patient was diagnosed with Parkinson’s disease shortly after testing. Assuming the disease was already present at the time of testing, we excluded them. Additionally, two control participants were excluded due to a Montreal Cognitive Assessment (MoCA) score below 26, which raised concerns about their classification as healthy.

### Ethics

The local ethics committee approved the study (Reference EA2/007/21). All participants provided written informed consent according to the Declaration of Helsinki.

### Clinical assessment

We evaluated the neurological disability and restrictions in daily life due to post-COVID-19 symptoms using the modified Rankin Scale (mRS).^38^ Comorbidities, medication, acute stage COVID-19 symptoms and current symptoms were recorded using a standardized protocol during in-person interviews.

### Neuropsychological assessment

Cognitive performance was assessed using a comprehensive test battery covering five cognitive domains (see Supplementary Table 1): (1) Memory and learning: verbal episodic memory [Rey Auditory Verbal Learning Test (RAVLT)^39^], visuospatial memory [Rey Osterrieth Complex Figure (ROCF)^40^], working memory (forward and backward digit span); (2) attention and alertness: tonic and phasic alertness, selective attention, and dual-tasking [Test of attentional performance battery (TAP)^41^], Trail-Making-Test A (TMT-A^42^); (3) executive function: Trail-Making-Test B (TMT-B^42^) and Stroop test^43^; (4) language: phonemic and semantic fluency [subtests of the Regensburg Word Fluency Test (RWT)^44^], and (5) logical thinking: Raven’s Progressive Matrices {German version [Leistungsprüfsystem (LPS), subtest 3^45^]. Furthermore, the MoCA was used as a screening tool for cognitive impairments.^46^

### Questionnaires

General health, health-related quality of life, and the level of independence were assessed using the Short Form Health Survey (SF-36),^47^ EQ-5D-5L,^48^ and a 0–10 scale of day-to-day independence evaluating 10 different life domains.^49^ We quantified levels of anxiety and depressive symptoms via the Hospital Anxiety and Depression Scale (HADS),^50^ Beck Depression Inventory II (BDI-II),^51^ and Beck Anxiety Inventory (BAI).^52^ Quality of sleep and daily sleepiness were surveyed (PSQI: Pittsburgh Sleep Quality Index)^53^ and ESS (Epworth Sleepiness Scale)^54^, as well as quantity and quality of fatigue (Fatigue Scale for Motor and Cognitive Functions (FSMC),^55^ Fatigue Severity Scale (FSS),^56^ Bell Score, and Canadian criteria for chronic fatigue syndrome/myalgic encephalomyelitis).^57^ The patients’ metamemory was examined using the Multifactorial Memory Questionnaire (MMQ).^58^

### MRI data acquisition

MRI data was acquired at the Berlin Centre of Advanced Neuroimaging (BCAN) using a 3 Tesla PRISMA scanner (Siemens, Erlangen) with a 64-channel head coil. As part of the protocol, a high-resolution structural T_1_-weighted sequence (3D-MPRAGE, TR = 1.900 ms, TE = 2.22 ms, T_1_ = 2.100 ms, voxel size 1 × 1 × 1 mm^3^, FOV = 256 × 256 × 192 mm) was acquired.

### MRI data analysis

Volumes of cortical and subcortical regions, as well as cortical thickness and surface area size were estimated using FreeSurfer, version 6.0.^59^ All images were visually checked and the pial surface was corrected if needed. Every volume, as well as cortical area and cortical thickness values, was adjusted for the total brain volume (*t*BV) within each participant using the formula: volume_adjusted_ = volume_observed_ − β (slope from *t*BV versus regional volume regression) × (*t*BV_observed_ − *t*BV_sample mean_). To assess general hippocampal as well as hippocampal subfield volumes, we ran the Freesurfer hippocampus subfield analysis.^60^ A visual quality check was also conducted for the hippocampal subfields in each participant. Cortical regions were analysed and compared in thickness, area size and volume using the Destrieux atlas.^61^

### Structural complexity analysis

Besides these classical neuroimaging measures, we applied a new method to estimate the structural complexity of brain regions. This approach rests on fractal analysis of high-resolution T1 MRI data and yields region-wise estimates of FD—a geometric measure of spatial scaling that expresses the irregularity of an object’s shape.^28,35^ Following previous approaches, we computed FD estimates with the calcFD toolbox for Matlab^28^ using the dilation algorithm for filled volumetric segmentations and default spatial scales and estimated FD values for each region of interest (ROI) defined by the Destrieux atlas.

### Statistical analysis

We performed all statistical analyses using R, version 4.1.2 and Matlab. Potential outliers were identified through visual inspection of boxplots. No extreme outliers were detected; therefore, none were excluded. Cognitive test scores, volumetric brain, cortex parcellation data and psychological survey scores were compared between patients and healthy controls using linear mixed models, including a random intercept for each matched pair. We used this method to accurately consider the person-to-person matching between patient and healthy control participant. The results were corrected for multiple testing using the Benjamini–Hochberg procedure to control the false discovery rate. Analyses of structural complexity were considered exploratory and were therefore reported without correction for multiple testing. Effect sizes were reported as fixed effect estimates (*b*) with 95% confidence intervals. For all linear mixed models, model fit was evaluated using information criteria, likelihood-ratio tests (LRT) and explained variance. Specifically, Akaike’s Information Criterion (AIC) and Bayesian Information Criterion (BIC) were compared between the full model (including the fixed effects of interest) and the corresponding null model (including only random effects). Significance of fixed effects was further assessed using LRT. To quantify the proportion of variance explained by fixed and random effects, marginal and conditional *R*^2^ values were calculated and are reported in the Supplementary Tables 1–3. To facilitate comparison across cognitive domains, raw test scores were converted to *z*-scores using the mean and standard deviation of the matched healthy control group. Correlation analyses were performed to assess associations between cognition, neuropsychiatric data and volumetric changes. Product-moment correlation was used for continuous, approximately normally distributed variables showing linear associations (assessed via Shapiro–Wilk tests and scatterplots). Spearman’s rank correlation was applied for non-normally distributed or ordinal variables, or when monotonic but non-linear relationships were expected. For variables with few distinct values or frequently tied ranks, such as the modified Rankin Scale, we used Kendall’s tau. Formal significance was defined at *P* < 0.05. Instances in which correlation results were not corrected for multiple testing are explicitly indicated. MRI and cognitive data were complete. There were missing values on the FSMC fatigue questionnaire for *n* = 6 patients and *n* = 7 healthy control participants. Analyses were conducted using only complete cases (pairwise exclusion).

## Results

### Clinical symptoms

The patients’ sample characteristics are presented in Table 1. Patients were tested at a median of 8 months (IQR 3 months) after SARS-CoV-2 infection. The acute disease stage lasted a median of 3 weeks (IQR 1 week). One patient experienced two COVID-19 episodes. 7/49 (14%) patients were hospitalized during their acute infection, of which 3/49 (6%) were admitted to the intensive care unit.

**Table 1:** Clinical characteristics

		Controls *n* (%)/median (IQR)	Patients *n* (%)/median (IQR)
Age [years]		42.5 (21)	44.0 (17)
Education [years]		17.0 (3)	16.0 (5)
Sex (in %)	female	39 (81%)	39 (80%)
	male	9 (19%)	10 (20%)
Acute COVID-19 duration [weeks]			3 (1)
Time since SARS-CoV-2 infection [months]			8 (3)
Modified Rankin Scale (mRS)			2 (0)
Unable to work entirely			22 (45%)
Unable to work to the pre-COVID-19 extent			11 (22%)
Patients’ pre-existing conditions			
Hypertension			7 (14%)
Asthma			6 (12%)
Allergies			4 (8%)
Hypothyroidism			4 (8%)
Infrequent migraines			2 (4%)
Coagulation disorders			2 (4%)
Cardiac arrhythmia			2 (4%)
Sleep apnea			1 (2%)
Restless legs syndrome			1 (2%)
Diabetes			1 (2%)
Patients’ newly onset conditions post-COVID-19			
Hypertension			3 (6%)
Asthma			2 (4%)
Hypothyroidism			1 (2%)
Cardiac arrhythmia			1 (2%)
Angina pectoris			1 (2%)
Rheumatoid arthritis			1 (2%)

mRS = Modified Rankin Scale.

The most common pre-existing conditions prior to COVID-19 were hypertension (7/49, 14%), asthma (6/49, 12%), allergies (4/49, 8%), and hypothyroidism (4/49, 8%) (full list see Table 1). After COVID-19, patients most frequently were diagnosed with newly onset hypertension (3/49, 6%) and asthma (2/49, 4%) (for a full list, see Table 1).

The most common self-reported cognitive complaints concerned concentration (80%), memory (62%), and word-finding capabilities (44%). Other neurological and psychiatric symptoms included the feeling of exhaustion (78%), tiredness (52%), headaches (38%), and feelings of high stress levels (36%), whereas somatic symptoms like dyspnoea (24%), muscle and joint pain (20%), chest pain (12%), and flu-like symptoms (10%) were less frequently reported. The median mRS of patients at the time of the study visit was 2 (IQR 0; Table 1).

### Cognitive performance

**Figure 1:**
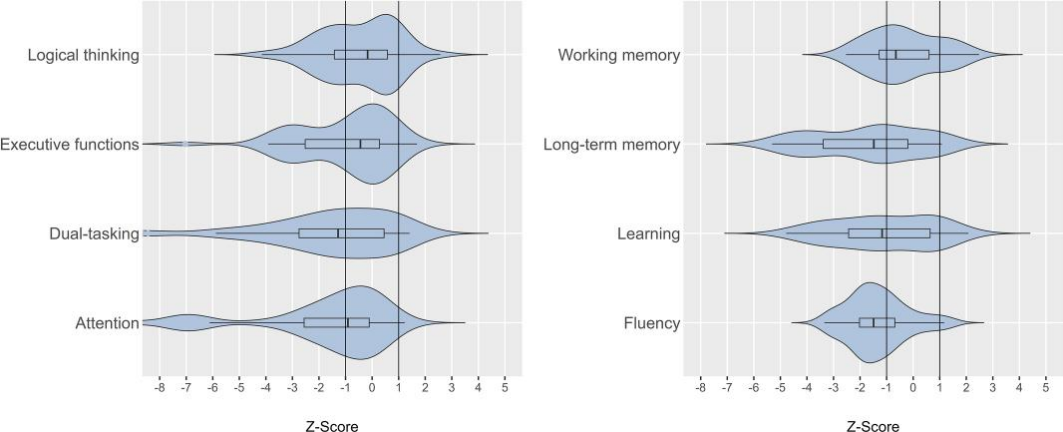
**Cognition in patients with post-COVID-19 condition (PCC; *N* = 49) compared to healthy controls (HC; *N* = 48).**  *Z*-scores are based on the mean and standard deviation of the control group and *Z* = −1 to 1 was considered normal cognitive performance. Working memory was assessed by Digit Span Forward (*P*-values adjusted for false discovery rate, pFDR = 0.168), long-term memory by the Rey Auditory Verbal Learning Test (RAVLT, trial 7, pFDR < 0.001), learning by RAVLT (trials 1–5, pFDR < 0.001), fluency by the Regensburger Word Fluency Test (RWT, s-words, pFDR < 0.001), logical thinking by the German Leistungsprüfsystem (LPS, pFDR = 0.067), executive functions by the Trail Making Test part B (TMT-B, pFDR = 0.002), and dual-tasking and attention by the Test of Attentional Performance (TAP; dual visual, pFDR = 0.001, and tonic alertness, pFDR < 0.001). All *P*-values were calculated using linear mixed models and corrected for multiple testing via Benjamini–Hochberg.

Patients performed significantly worse than the healthy control participants for tests of attention (tonic alertness, *b* = 52.4 ms, 95% confidence interval (CI) [29.3, 75.6], pFDR < 0.001), executive function (TMT-B, *b* = 14.8 s, 95% CI [5.7, 24.0], pFDR = 0.002), fluency (phonemic, *b* = −5.1, 95% CI [−6.9, −3.3] and semantic, *b* = −5.0, 95% CI [−7.3, −2.8], both pFDR < 0.001), verbal learning (RAVLT, trials 1–5, *b* = −6.3, 95% CI [−9.4, −2.9], pFDR < 0.001), verbal memory (RAVLT, trial 7, *b* = −2.7, 95% CI [−3.8, −1.7], pFDR < 0.001), and visuospatial memory (ROCF, delayed recall, *b* = −4.8, 95% CI [−7.1, −2.5], pFDR < 0.001) (Fig. 1; see Supplementary Table 1 for all test results). No significant group differences were observed for logical thinking capabilities (LPS pFDR = 0.067) and short-term memory (digit span forward pFDR = 0.168; Supplementary Table 1).

#### Association with clinical symptoms

**Figure 2:**
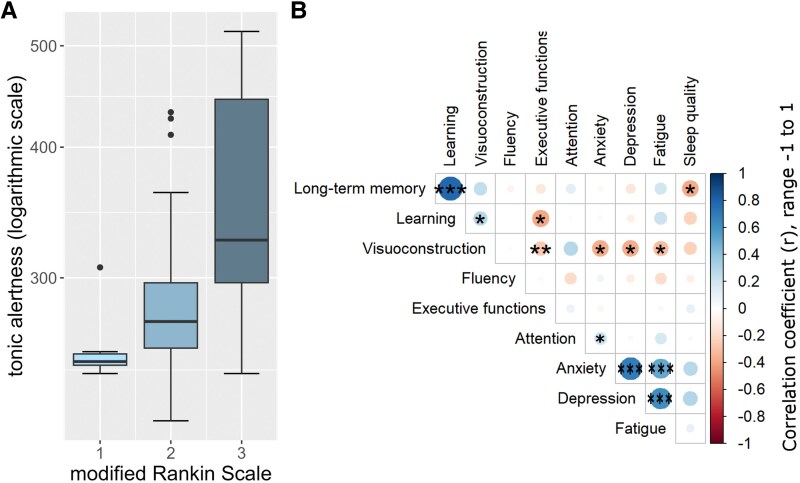
**Correlation between everyday functioning and attention and correlation plot of cognitive outcomes and neuropsychiatric data in patients with post-COVID-19 condition (PCC; *N* = 49). (A)** Higher scores on the modified Rankin Scale (mRS; greater disability) were associated with significantly longer reaction times (Kendall’s *τ* = 0.417, *P*-values adjusted for false discovery rate, pFDR < 0.001). (**B)** Correlation of cognitive outcomes and neuropsychiatric data. Long-term memory was assessed by the Rey Auditory Verbal Learning Test (RAVLT, trial 7), learning by RAVLT (trials 1–5), visuoconstruction by the Rey–Osterrieth Complex Figure Test (ROCF), fluency by the Regensburger Word Fluency Test (RWT, s-words), executive functions by the Trail Making Test part B (TMT-B), attention by the Test of Attentional Performance (TAP; tonic alertness), anxiety by the Beck Anxiety Inventory (BAI), depressive symptoms by the Beck Depression Inventory-II (BDI-II), fatigue by the Fatigue Scale for Motor and Cognitive Functions (FSMC), sleep quality by the Pittsburgh Sleep Quality Index (PSQI). Correlations were calculated using Pearson correlation coefficients, with *P*-values adjusted for false discovery rate. Symbols indicate statistical significance: *P < 0.05; **P < 0.01; ***P < 0.001.

Cognitive performance was not associated with the duration of the acute phase of COVID-19, the number of acute symptoms, or the time elapsed since the acute infection (absolute range *r* = 0.007 to *r* = 0.278, all *P* > 0.050). Higher neurological disability (higher mRS scores) significantly correlated with worse tonic alertness (*τ* = 0.417, pFDR < 0.001, Fig. 2; Supplementary Table 4).

#### Association with neuropsychiatric symptoms

Patients had significantly higher levels of anxiety, depressive symptoms, fatigue, daytime sleepiness, and worse quality of sleep, as well as worse subjective memory functioning and memory satisfaction (Supplementary Table 3). Worse tonic alertness performance correlated with higher anxiety levels (*τ* = 0.260, pFDR = 0.016). Reduced short-term visuospatial memory performance was associated with higher levels of anxiety (BAI: *τ* = −0.250, pFDR = 0.021), depressive symptoms (BDI-II: *τ* = −0.255, pFDR = 0.020), and fatigue (FSMC: *τ* = −0.220, *P* = 0.041, Fig. 2). Furthermore, worse sleep quality (PSQI) correlated with worse performance in long-term verbal memory (*τ* = −0.277, pFDR = 0.016; Fig. 2).

#### Subjective memory satisfaction versus actual memory performance

Several visuospatial and verbal mnemonic task results of patients correlated with their reported memory satisfaction (ROCF: *ρ* = 0.507, *P* < 0.001, RAVLT: *ρ* = 0.356, pFDR = 0.019).

### Quality of life

Patients reported significantly lower quality of life (Supplementary Table 3). General health-related quality of life and emotional wellbeing were significantly associated with fatigue, depressive symptoms and anxiety (general health × FSMC: *τ* = −0.292, pFDR = 0.009; general health × BDI: *τ* = −0.272, pFDR = 0.015; emotional wellbeing × FSMC: *τ* = −0.323, pFDR = 0.004; emotional wellbeing × BAI: *τ* = −0.469, pFDR < 0.001, emotional wellbeing × BDI: *τ* = −0.644, pFDR < 0.001). Cognitive performance, even though significantly impaired, did not correlate with quality of life (absolute range *τ* = 0.004 to 0.166, all *P* > 0.05).

### Structural MRI results

**Figure 3:**
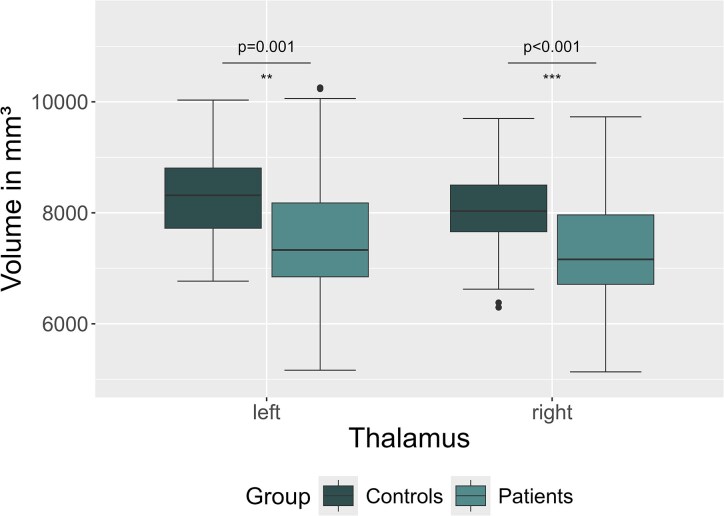
**Thalamic volume changes in patients with post-COVID-19 condition (PCC, *N* = 49).** Patients with PCC have significantly smaller thalamic volumes compared to healthy control participants. Symbols indicate statistical significance: **P < 0.01; ***P < 0.001. *P*-values were calculated using linear mixed models and corrected for multiple testing via Benjamini–Hochberg.

Patients had lower thalamic volumes compared to control participants (left: patients, 7621.9 (±1130.0) mm^3^ versus controls, 8293.9 (±836.5) mm^3^, *b* = −672.0 mm^3^, 95% CI [−1068.1, −275.9], pFDR = 0.001; right: patients, 7363.2 (±935.3) mm^3^ versus controls, 8016.7 (±769.6), *b* = −653.5 mm^3^, 95% CI [−994.5, −312.4], pFDR < 0.001, Fig. 3). There was no significant difference in volume in other subcortical regions (Supplementary Table 2). Volumetry of hippocampal subfields did not indicate significant differences between patients and controls. Furthermore, there were no significant group differences in cortical thickness, cortical area size or cortical volume between patients and the control participants after Benjamini–Hochberg correction for multiple testing.

There were no significant correlations between patients’ thalamic volume and cognitive test performances. In an exploratory analysis of ROIs with significant group differences, we found that patients’ lower left thalamic volumes correlated with more pronounced daytime sleepiness (*ρ* = −0.32, *P* = 0.040).

#### Structural complexity analysis

**Figure 4:**
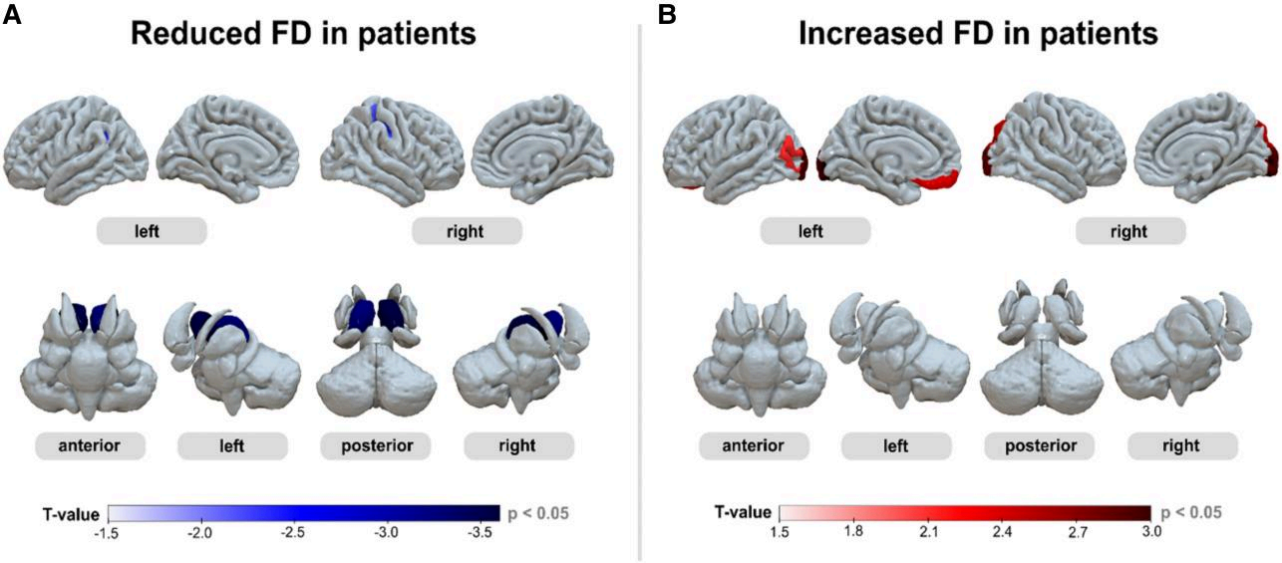
**Patients with post-COVID-19 condition (*N* = 49) show bidirectional changes of structural brain complexity. (A)** Significantly reduced fractal dimensionality (FD) in patients was found in the left and right thalamus. (**B)** Significant increases were found in the left gyrus rectus, left middle occipital gyrus, right superior occipital gyrus, and left and right occipital pole. *P*-values were calculated using two-sample *t*-test.

Patients with PCC showed bidirectional changes in structural brain complexity compared to the healthy control participants (Fig. 4). The strongest *decreases* in complexity were observed in the thalamus, both on the left (*t* = −3.4, *P* = 0.001) and on the right (*t* = −3.6, *P* = 5 × 10^−4^). However, patients also presented a spatial cluster of *increased* complexity (Fig. 4, right) that involved the left gyrus rectus (*t* = 2.4, *P* = 0.019), the left middle occipital gyrus (*t* = 2.1, *P* = 0.037), the right superior occipital gyrus (*t* = 2.5, *P* = 0.016) as well as the occipital pole bilaterally (left: *t* = 2.9, *P* = 0.004; right: *t* = 2.9, *P* = 0.005). Moreover, similar bidirectional changes were also detected in hippocampal subfield analysis, which showed decreased complexity in the CA1 region on the left (*t* = −2.3, *P* = 0.023) as well as pronounced increases in the fimbria bilaterally (left: *t* = 4.1, *P* = 1 × 10^−4^; right: *t* = 3.3, *P* = 0.001).

**Figure 5:**
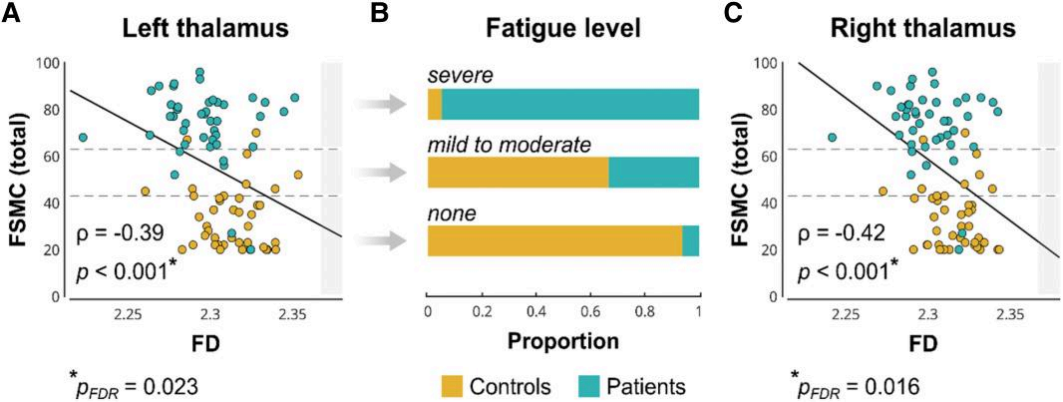
**Lower structural complexity of the thalamus is associated with higher fatigue scores in patients with post-COVID-19 condition (PCC, *N* = 42) and healthy control participants (HC, *N* = 41).** Associations between fractal dimensionality (FD) of the left **(A)** and right thalamus **(C)** and total scores on the Fatigue Scale for Motor and Cognitive Functions (FSMC) are shown. Each data point describes one patient or HC. Panel **B** summarizes the proportions of patients with PCC and HC showing no fatigue (FSMC < 43), mild to moderate fatigue (FSMC = 43–62), and severe fatigue (FSMC > 62). No *y*-axis is shown, as the bars are directly labelled with their values. Correlations were calculated using Spearman correlation coefficients, with *P*-values adjusted for false discovery rate.

Additionally, lower structural complexity of the thalamus was related to higher fatigue scores on the FSMC (Fig. 5), both on the left (*ρ* = −0.39, *P* = 2.3 × 10^−4^, p_FDR_ = 0.023) and on the right (*ρ* = −0.42, *P* = 7.5 × 10^−5^, p_FDR_ = 0.016). Besides these thalamic complexity changes, the strongest associations between FSMC scores and structural complexity were observed in the middle frontal sulcus on the right (*ρ* = −0.36, *P* = 7.7 × 10^−4^, p_FDR_ = 0.040) and the CA1 hippocampal subfield on the left (*ρ* = −0.36, *P* = 6.8 × 10^−4^, p_FDR_ = 0.040).

## Discussion

In the current study, we found that post-COVID-19 patients with subjective cognitive decline had objective cognitive deficits affecting attention, long-term memory, executive functions and verbal fluency. In addition, patients had higher levels of fatigue, depressive symptoms, anxiety, daytime sleepiness and worse quality of sleep. They were negatively impacted in their ability to work and had a lower quality of life, which was associated with fatigue severity. Furthermore, we identified significantly reduced thalamic volumes and reduced thalamic structural complexity bilaterally in post-COVID-19 patients. The reduction of thalamic complexity correlated with increased fatigue severity in patients and controls.

Patients with PCC showed substantial cognitive deficits across all cognitive domains except short-term memory and logical thinking. These deficits were not associated with the severity of the acute infection, which is in line with previous studies that similarly found no association between acute COVID-19 severity and cognitive outcomes.^62^ Considering our patients’ young average age and overall good pre-COVID-19 health status, these deficits entail major impairments in both their personal and professional lives. Indeed, 45% of patients were entirely unable to work and 22% were unable to perform their usual level of work due to post-COVID-19 cognitive problems and fatigue. Furthermore, most patients found tasks of daily life more difficult, ranging from ‘needing more time during household chores’ to ‘being unable to look after themselves entirely’. These impairments were associated with difficulties in concentrating, planning, remembering or other cognitive tasks. We assessed everyday functioning and independence and found it to significantly correlate with patients’ attention performance. These findings indicate the importance of cognitive wellbeing and thus cognitive assessments in patients with PCC, regardless of the severity of the acute infection or the duration since the acute disease.

Prior interview-based analyses in larger post-COVID-19 samples found cognitive impairment to be one of the most prevalent subjective long-term symptoms.^5,6^ Here, we show that such subjective impairments correspond to objective deficits when using an extensive neuropsychological assessment. In addition, subjective memory satisfaction correlated with objective memory performance. Our finding that deficits affect nearly all cognitive domains is consistent with earlier research assessing memory, attention and executive function.^10,63,64^ However, many studies used normative data collected before the pandemic that is thus not affected by general pandemic-related stressors. Here, we show that global deficits can be observed even compared to age-, sex- and education-matched control participants assessed in the same setting during the pandemic.

We also observed significantly increased levels of depressive symptoms, anxiety, sleep-related problems and fatigue in patients with PCC. Furthermore, patients reported significantly reduced health-related quality of life, which was associated with higher levels of fatigue and depressive symptoms, consistent with earlier data from post-COVID-19 clinics.^65^ Importantly, we found depressive symptoms, anxiety and fatigue—but not cognitive performance—to be the main drivers for health-related quality of life and emotional wellbeing even though cognitive impairment affected almost all domains.

It has also been suggested that cognitive impairment in PCC might be the result of psychological stress and/or fatigue. However, recent analyses of large cohort studies show that cognitive impairment and fatigue are two distinct phenomena and affect different patient groups with PCC,^66^ with women and younger patients more frequently suffering from fatigue and older and male patients more frequently having cognitive deficits. Moreover, cognitive complaints have been shown to persist longer than psychological symptoms following SARS-CoV-2 infections.^67^ Taken together, our results highlight the high prevalence of cognitive impairment, fatigue and neuropsychiatric symptoms, the complex interplay of these symptoms and their major detrimental effects on patients’ quality of life, emotional wellbeing and ability to work.

Our MRI analyses identified relevant structural brain changes in patients with PCC. Patients had reduced thalamic volumes in both hemispheres compared to well-matched control participants. There was a significant correlation between patients’ lower left thalamic volumes and increased daytime sleepiness. This association is in line with previous research showing a link between thalamic integrity and the sleep–wake cycle regulation.^68,69^ However, our results are to be interpreted with caution as the observed effect size was relatively small and exploratory analyses were not corrected for multiple testing. In addition, daytime sleepiness was assessed with self-report questionnaires. Future studies should confirm this association in larger studies with both patient-reported measures and objective assessments of nightly sleep and daytime sleepiness. In contrast to earlier studies that reported cortical changes in the olfactory cortex and limbic system,^18-20,24,70^ we found no significant differences in cortical thickness. In addition, this is the first study applying FD analyses in a cohort of post-COVID-19 patients. We observed reduced structural complexity in the left and right thalamus, with lower complexity correlating with higher fatigue scores across patients and control participants. Of note, although severe fatigue was more common in patients, mild-to-moderate FSMC scores were in fact more prevalent in controls (Fig. 5, middle), suggesting that the observed thalamus–fatigue association reflects fatigue pathophysiology in general rather than a PCC-specific aetiology. Indeed, our observations are in line with robust evidence linking thalamus and basal ganglia damage as well as reduced oxygen perfusion to high levels of fatigue in patients with PCC^26,71^ and with other neurological disorders including multiple sclerosis.^72-74^ Together with the central role of the thalamus as a cortex gateway and its involvement in the management of alertness, a shared pathophysiological pathway for fatigue involving damage of the thalamus appears plausible.

Moreover, structural complexity analysis detected additional alterations of brain morphology (e.g. occipital lobes, hippocampal subfields) that remained undetected in standard volumetry, supporting the potential of fractal analysis as a useful tool to capture structural brain changes in neurological disorders.^27^ Nonetheless, future studies in larger cohorts and comparative analyses in other neurological disorders are needed to better understand the specificity of these findings. Moreover, we here limited shape analysis to FD because of promising findings in other neurological conditions^29-31^ and because it is estimated from the same input data as standard volumetry and thus represents the most direct comparison to the latter.^27,28,35^ However, complementary approaches such as surface-based morphometry, deformation-based morphometry or SPHARM-PDM may capture different aspects of brain shape, such that future studies should directly compare these techniques in PCC.

Our study has several limitations: (1) Given the cross-sectional design, causal directions regarding the observed associations and a potential reversibility of imaging alterations cannot be determined. (2) As in many studies, our sample is characterized by a high average education level and a skewed gender distribution towards women. Our results may therefore not be generalizable to all sociodemographic groups. However, PCC is more common in women,^37,66^ such that our gender distribution is representative for PCC. In addition, careful age-, sex- and education-based person-to-person matching reduced the risk of bias for group comparisons. (3) Our morphological assessment combined volumetry with FD analysis but did not incorporate additional shape-sensitive frameworks. Strengths of our study include (1) an extensive neuropsychological assessment and MR imaging protocol; (2) an assessment of neuropsychiatric symptoms, fatigue, and quality of life using standardized questionnaires; (3) well-matched healthy control participants that were exposed to the same general pandemic-related stressors such as confinement, social isolation and financial insecurity as patients with PCC; (4) the prospective study design that ensured similar testing conditions for all participants, and (5) the use of advanced imaging analyses including assessment of FD.

In conclusion, subjective cognitive complaints of patients with PCC reflected deficits across almost all cognitive domains in standardized tests. In addition, patients with PCC had increased levels of fatigue, depressive symptoms, anxiety and sleep problems resulting in significantly reduced quality of life and impaired ability to work. Imaging analyses identified reduced volumes and reduced complexity of the thalamus correlating with higher levels of fatigue and implicating the thalamus as a key brain region involved in fatigue pathophysiology. These data highlight the complex structural brain findings and neuropsychiatric symptoms in patients with PCC. Longitudinal studies are now required to assess whether these symptoms respond to symptomatic treatment and whether associated brain structural changes may be reversible.

## Supplementary Material

fcag099_Supplementary_Data

## Data Availability

Data supporting the findings of this study are available from the corresponding author upon reasonable request. The code is available at https://osf.io/aym9e/overview?view_only=caf7fa45f07b46f6a41e050a4a270861.
