# Supplementary material for: Association of structural brain changes with cognitive deficits and fatigue in patients with post-COVID-19 condition
Source: Brain Commun. 2026 Mar 18;8(2):fcag099. doi: 10.1093/braincomms/fcag099 (PMC13049549; doi:10.1093/braincomms/fcag099)
Supplement: fcag099_Supplementary_Data [file fcag099_supplementary_data.docx]

**SUPPLEMENTARY TABLES**

**Supplementary table 1. Cognitive outcome in patients and control participants.** (MoCa: Montreal Cognitive Assessment, RAVLT: Rey Auditory Verbal Learning Test, ROCF: Rey-Osterrieth Complex Figure, TAP: test for attentional performance battery, TMT: Trail-Making-Test, LPS: ‘Leistungsprüfsystem’ (Raven’s Progressive Matrices), *p<0.01, **p<0.001, ***p<0.001 (all FDR-corrected))

|  | | **Controls**   mean (±SD) | **Patients**   mean (±SD) | **Fixed effects estimate (b, 95% CI)** | **p-value** | **Marginal/ Conditional R²** |
| --- | --- | --- | --- | --- | --- | --- |
| *Screening* | | | | | |  |
| **MoCa** |  | 28.4 (±1.3) | 26.7 (±2.6) | b=-1.7 [-2.5, -1.0] | <0.001 *** | 0.16/ 0.24 |
| *Memory* | | | | | |  |
| **RAVLT** | trial 1 | 7.3 (±2.3) | 6.5 (±1.9) | b=-0.8 [-1.6, 0.0] | 0.066 | 0.03/ 0.04 |
|  | trial 5 | 14.2 (±1.2) | 12.8 (±2.1) | b=-1.4 [-2.1, -0.7] | <0.001 *** | 0.14/ 0.16 |
|  | sum (1-5) | 58.5 (±7.4) | 52.2 (±10.4) | b=-6.3 [-9.4, -2.9] | <0.001 *** | 0.11/ 0.26 |
|  | interference | 7.2 (±2.2) | 5.8 (±1.9) | b=-1.4 [-2.2, -0.5] | 0.001 ** | 0.10/ 0.10 |
|  | trial 6 | 13.2 (±2.0) | 10.7 (±3.5) | b=2.5 [-3.6, -1.4] | <0.001 *** | 0.16/ 0.26 |
|  | trial 7 (recall) | 13.3 (±1.9) | 10.6 (±3.1) | b=-2.7 [-3.8, -1.7] | <0.001 *** | 0.22/ 0.22 |
|  | recognition (uncorrected) | 13.5 (±1.8) | 13.6 (±1.7) | b=-0.6 [-1.2, -0.3] | 0.040 * | 0.04/ 0.04 |
|  | recognition (corrected) | 14.0 (±1.4) | 11.7 (±3.2) | b=-2.3 [-3.2, -1,3] | <0.001 *** | 0.18/ 0.20 |
| **ROCF** | copy | 35.4 (±0.9) | 35.1 (±1.2) | b=-0.3 [-0.6, 0.2] | 0.332 | 0.01/ 0.01 |
|  | immediate recall | 25.5 (±5.3) | 21.2 (±7.1) | b=-4.3 [-6.8, -1.8] | <0.001 *** | 0.11/ 0.11 |
|  | delayed recall | 25.3 (±5.2) | 20.5 (±6.3) | b=-4.8 [-7.1, -2.5] | <0.001 *** | 0.15/ 0.15 |
| **Digit Span** | forward | 8.0 (±2.0) | 7.5 (±2.1) | b=-0.5 [-1.3, 0.2] | 0.168 | 0.02/ 0.19 |
|  | backward | 7.4 (±2.0) | 6.5 (±2.0) | b=-0.9 [-1.6, -0.2] | 0.019 * | 0.05/ 0.17 |
| *Attention* | | | | | |  |
| **TAP** | tonic alertness | 251.1 (±40) ms | 303.5 (±73) ms | b=52.4 [29.3, 75.6] | <0.001 *** | 0.17/ 0.21 |
|  | phasic alertness | 256.4 (±37) ms | 296.7 (±66) ms | b=40.3 [19.7, 60.8] | <0.001 *** | 0.13/ 0.22 |
|  | selective attention | 495.1 (±93) ms | 560.5 (±116)ms | b=65.3 [23.4, 107.2] | 0.003 ** | 0.09/ 0.09 |
|  | dual task auditory | 536.8 (±86) ms | 656.6 (±183) ms | b=119.8 [62.8, 176.8] | <0.001 *** | 0.15/0.15 |
|  | dual task visual | 723.6 (±71) ms | 807.9 (±181) ms | b=83.5 [35.4, 131.7] | 0.001 ** | 0.09/ 0.31 |
| **TMT** | A | 27.4 (±11.0) s | 34.3 (±11.1) s | b=6.9 [2.8, 10.9] | 0.002 ** | 0.09/ 0.23 |
| *Executive functions* | | | | | |  |
| **TMT** | B | 54.8 (±20.8) s | 69.6 (±25.4) s | b=14.8 [5.7, 24.0] | 0.002 ** | 0.09/ 0.13 |
| **Stroop** |  | 100.1 (±23.7) s | 125.0 (±35.4) s | b=24.9 [12.9, 36.9] | <0.001 *** | 0.15/ 0.15 |
| *Language* | | | | | |  |
| **Fluency** | phonemic (s-words) | 19.6 (±4.8) | 14.5 (±4.2) | b=-5.1 [-6.9, -3.3] | <0.001 *** | 0.25/ 0.25 |
|  | semantic (animals) | 29.2 (±5.7) | 24.1 (±5.7) | b=-5.0 [-7.3, -2.8] | <0.001 *** | 0.17/ 0.17 |
| *Logical Thinking* | | | | | |  |
| **LPS** | subtest 3 | 29.7 (±4.9) | 27.8 (±5.8) | b=-1.9 [-3.9, 0.1] | 0.067 | 0.03/ 0.13 |

**Supplementary table 2. Volumetric outcome of subcortical areas in patients and control participants.** (Significance of p-values in brackets were not significant after Benjamini-Hochberg correction, *p<0.01, **p<0.001, ***p<0.001 (FDR-corrected))

|  | | **Controls**  mean volume   in mm³ (±SD) | **Patients**  mean volume   in mm³ (±SD) | **Fixed effects estimate**  **(b, 95% CI)** | **p-value** | **Marginal/ Conditional R²** |
| --- | --- | --- | --- | --- | --- | --- |
| **Amygdala** | left | 1497.2 (±164.8) | 1577.3 (±239.7) | b=80.7 [3.0, 158.0] | (0.043) | 0.04/ 0.16 |
|  | right | 1626.9 (±176.8) | 1714.8 (±254.0) | b=87.9 [0.7, 175.1] | (0.049) | 0.04/ 0.04 |
| **Caudate ncl.** | left | 3583.4 (±450.4) | 3490.9 (±470.4) | b=-98.3 [-268.3, 73.6] | 0.256 | 0.01/ 0.15 |
|  | right | 3722.0 (±515.3) | 3613.1 (±511.3) | b=-119.0 [-291.5, 54.9] | 0.176 | 0.01/ 0.30 |
| **Hippocampus** | left | 3567.9 (±341.6) | 3538.3 (±360.3) | b=-29.6 [-169.3, 110.1] | 0.676 | 0.00/ 0.00 |
|  | right | 3626.99 (±358.4) | 3619.1 (±393.5) | b=-7.9 [-157.7, 141.9] | 0.917 | 0.00/ 0.00 |
| **Accumbens ncl.** | left | 471.6 (±91.2) | 452.7 (±94.3) | b=-17.5 [-47.7, 12.6] | 0.252 | 0.01/ 0.35 |
|  | right | 550.1 (±89.7) | 545.4 (±86.5) | b=-3.5 [-33.1, 26.0] | 0.817 | 0.00/ 0.30 |
| **Pallidum** | left | 2118.7 (±235.5) | 2065.0 (±211.1) | b=-53.7 [-142.6, 35.2] | 0.235 | 0.01/ 0.01 |
|  | right | 1954.0 (±240.2) | 1904.0 (±223.7) | b=-49.6 [-138.1, 38.6] | 0.267 | 0.00/ 0.38 |
| **Putamen** | left | 4798.2 (±583.6) | 4673.1 (±592.2) | b=-122.1 [-306.8, 62.2] | 0.192 | 0.01/ 0.40 |
|  | right | 4809.3 (±624.4) | 4754.2 (±576.6) | b=-50.0 [-240.6, 140.0] | 0.602 | 0.00/ 0.38 |
| **Thalamus** | left | 8293.9 (±836.5) | 7621.9 (±1130.0) | b=-672.0 [-1068.1, -275,9] | 0.001 ** | 0.11/ 0.11 |
|  | right | 8016.7 (±769.6) | 7363.2 (±935.3) | b=-653.5 [-994.5, -312.4] | <0.001 *** | 0.13/ 0.13 |

**Supplementary table 3. Self-reported neuropsychiatric outcomes in patients and controlparticipants.** (SF-36: Short Form Health Survey, EQ-5D-5L: EuroQuol 5 dimensions 5 levels survey, EQ-VAS: EuroQuol visual analogue scale, HADS: Hospital Anxiety and Depression Scale, BDI-II: Beck Depression Inventory 2, BAI: Beck Anxiety Inventory , FSMC: Fatigue Scale for Motor and Cognitive Functions, FSS: Fatigue Severity Scale, CFS: Chronic Fatigue Syndrome, PSQI: Pittsburgh Sleep Quality Index, ESS: Epworth Sleepiness Scale, MMQ: Multifactorial Memory Questionnaire, **p<0.001, ***p<0.001 (all FDR-corrected))

|  |  | **Controls**   mean (±SD) | **Patients**   mean (±SD) | **Fixed effects estimate**   **(b, 95% CI)** | **p-value** | **Marginal/ Conditional R²** |
| --- | --- | --- | --- | --- | --- | --- |
| *General health and independency* | | | | | |  |
| **SF-36** | physical functioning | 95.4 (±8.5) | 60.8 (±23.0) | b=-34.6 [-41.3, -27.9] | <0.001 *** | 0.52/ 0.56 |
|  | limitations physical role | 94.3 (±18.0) | 16.9 (±28.7) | b=-77.4 [-86.8, -68.0] | <0.001 *** | 0.73/ 0.76 |
|  | limitations emotional role | 82.7 (±25.3) | 37.1 (±33.4) | b=-44.9 [-58.2, -31.8] | <0.001 *** | 0.29/ 0.46 |
|  | energy | 62.9 (±20.0) | 26.2 (±17.1) | b=-36.7 [-44.4, -29.1] | <0.001 *** | 0.50/ 0.52 |
|  | emotional wellbeing | 78.3 (±16.3) | 61.2 (±16.7) | b=-17.1 [-23.9, -10.4] | <0.001 *** | 0.22/ 0.23 |
|  | social functioning | 89.4 (±14.9) | 48.0 (±19.1) | b=-41.4 [-50.6, -32.2] | <0.001 *** | 0.47/ 0.47 |
|  | pain | 87.3 (±16.3) | 56.9 (±23.9) | b=-30.4 [-41.0, -19.8] | <0.001 *** | 0.26/ 0.26 |
|  | general health | 75.4 (±18.4) | 47.1 (±17.2) | b=-28.3 [-35.6, -21.0] | <0.001 *** | 0.39/ 0.39 |
| **EQ-5D-5L** | mobility | 1.1  (±0.3) | 1.9  (±0.9) | b=0.8 [0.5, 1.1] | <0.001 *** | 0.27/ 0.27 |
|  | selfcare | 1.0  (±0.1) | 1.3  (±0.6) | b=0.3 [0.1, 0.4] | <0.001 *** | 0.11/ 0.11 |
|  | usual activities | 1.2  (±0.5) | 3.0  (±1.1) | b=1.9 [1.5, 2.2] | <0.001 *** | 0.56/ 0.59 |
|  | pain/ discomfort | 1.4  (±0.6) | 2.3  (±0.8) | b=0.9 [0.6, 1.2] | <0.001 *** | 0.29/ 0.31 |
|  | anxiety/ depression | 1.2  (±0.5) | 2.0  (±0.9) | b=0.8 [0.5, 1.1] | <0.001 *** | 0.26/ 0.29 |
| **EQ-VAS** | overall health | 85.6  (±9.0) | 55.1 (±17.9) | b=-30.6 [-36.0, -25.0] | <0.001 *** | 0.55/ 0.61 |
| **Independency** |  | 9.7 (±0.5) | 6.7 (±1.7) | b=-3.0 [-3.7, -2.4] | <0.001 *** | 0.50/ 0.53 |
| *Depressive symptoms and anxiety* | | | | | |  |
| **HADS** | total | 4.5 (±4.9) | 13.8 (±7.8) | b=9.3 [6.6, 11.9] | <0.001 *** | 0.35/ 0.35 |
|  | anxiety | 2.3 (±2.7) | 7.0 (±4.0) | b=4.6 [3.3, 6.0] | <0.001 *** | 0.33/ 0.33 |
|  | depression | 1.7 (±2.5) | 6.8 (±4.6) | b=5.1 [3.6, 6.6] | <0.001 *** | 0.33/ 0.33 |
| **BDI-II** |  | 4.5 (±5.1) | 16.4 (±8.1) | b=11.9 [9.0, 14.7] | <0.001 *** | 0.44/ 0.47 |
| **BAI** |  | 3.2 (±4.5) | 14.0 (±7.9) | b=10.7 [8.0, 13.4] | <0.001 *** | 0.42/ 0.42 |
| *Fatigue* | | | | | |  |
| **FSMC** | total | 33.8 (±13.4) | 73.5 (±15.1) | b=39.5 [33.9, 45.3] | <0.001 *** | 0.66/ 0.72 |
|  | cognitive | 16.8 (±7.5) | 37.1 (±8.1) | b=20.2 [17.1, 23.4] | <0.001 *** | 0.63/ 0.69 |
|  | motor | 17.0 (±6.5) | 36.4 (±8.1) | b=19.4 [16.4, 22.3] | <0.001 *** | 0.64/ 0.71 |
| **FSS** |  | 22.1 (±9.9) | 51.9 (±11.7) | b=29.7 [25.4, 34.0] | <0.001 *** | 0.66/ 0.72 |
| **Bell-Score** |  | 97.6 (±5.8) | 50.5 (±17.6) | b=-47.1 [-52.8, -41.4] | <0.001 *** | 0.77/ 0.77 |
| **Canadian criteria** | for CFS | 0.0 (±0.2) | 0.3 (±0.5) | b=0.3 [0.1, 0.4] | <0.001 *** | 0.13/ 0.13 |
| *Sleep* | | | | | |  |
| **PSQI** |  | 4.3 (±2.3) | 8.0 (±3.8) | b=3.7 [2.5, 4.9] | <0.001 *** | 0.27/ 0.46 |
| **ESS** |  | 5.1 (±2.5) | 10.5 (±5.5) | b=5.4 [3.6, 7.2] | <0.001 *** | 0.29/ 0.31 |
| *Metamemory* | | | | | |  |
| **MMQ** | satisfaction | 60.9 (±9.5) | 32.3 (±13.4) | b=-28.7 [-33.4, -23.9] | <0.001 *** | 0.62/ 0.62 |
|  | functioning | 66.0 (±9.7) | 41.2 (±14.9) | b=-24.9 [-30.0, -19.8] | <0.001 *** | 0.51/ 0.51 |
|  | strategies | 25.9 (±10.4) | 33.3 (±10.8) | b=7.4 [3.0, 11.8] | 0.001 ** | 0.11/ 0.11 |

**Supplemetary table 4. Correlation between modified Rankin Scale (mRS) and exemplary cognitive tests**. Correlations via Kendall Tau, p-values corrected via Benjamini-Hochberg. (RAVLT: Rey Auditory Verbal Learning Test, TAP: test of attentional performance battery, TMT: Trail-Making-Test, *p<0.01, **p<0.001, ***p<0.001)

| **Cognitive domain** | **Correlation** | **τ** | **p** |
| --- | --- | --- | --- |
| Long-term memory | RAVLT trial 7 x mRS | 0.106 | 0.378 |
| Learning | RAVLT trial 1-5 x mRS | 0.081 | 0.484 |
| Fluency | phonemic fluency x mRS | -0.158 | 0.18 |
| Executive functions | TMT B x mRS | 0.085 | 0.459 |
| Attention | TAP tonic alertness x mRS | 0.417 | <0.001 *** |
